# Gastrectomy compared to oesophagectomy for Siewert II and III gastro-oesophageal junctional cancer in relation to resection margins, lymphadenectomy and survival

**DOI:** 10.1038/s41598-017-18005-6

**Published:** 2017-12-19

**Authors:** Joonas H. Kauppila, Karl Wahlin, Jesper Lagergren

**Affiliations:** 1 Upper Gastrointestinal Surgery, Department of Molecular medicine and Surgery, Karolinska Institutet, Karolinska University Hospital, 17176 Stockholm, Sweden; 2 Cancer and Translational Medicine Research Unit, Medical Research Center Oulu, University of Oulu, Oulu University Hospital, Oulu, Finland; 3 Division of Cancer Studies, King’s College London and Guy’s and St Thomas’ NHS Foundation Trust, London, England

## Abstract

It is unclear whether gastrectomy or oesophagectomy offer better outcomes for gastro-oesophageal junction (GOJ) cancer. A total of 240 patients undergoing total gastrectomy (n = 85) or oesophagectomy (n = 155) for Siewert II-III GOJ adenocarcinoma were identified from a Swedish prospective population-based nationwide cohort. The surgical approaches were compared in relation to non-radical resection margins (main outcome) using multivariable logistic regression, providing odds ratios (ORs) and 95% confidence intervals (CIs), mean number of removed lymph nodes with standard deviation (SD) using ANCOVA, assessing mean differences and 95% CIs, and 5-year mortality using Cox regression estimating hazard ratios (HRs) and 95% CIs. The models were adjusted for age, sex, comorbidity, tumour stage, and surgeon volume. The non-radical resection rate was 15% for gastrectomy and 14% for oesophagectomy, and the adjusted OR was 1.61 (95% CI 0.68–3.83). The mean number of lymph nodes removed was 14.2 (SD ± 9.6) for gastrectomy and 14.2 (SD ± 10.4) for oesophagectomy, with adjusted mean difference of 2.4 (95% CI-0.2–5.0). The 5-year mortality was 76% following gastrectomy and 75% following oesophagectomy, with adjusted HR = 1.07 (95% CI 0.78–1.47). Gastrectomy and oesophagectomy for Siewert II or III GOJ cancer seem comparable regarding tumour-free resection margins, lymph nodes removal, and 5-year survival.

## Introduction

The curative treatment of adenocarcinoma of the gastro-oesophageal junction (GOJ) typically includes surgical resection. Multimodality treatment and centralization of surgery has improved the 5-year survival rates^1,2^, but there is an on-going debate about the optimal surgical approach for GOJ cancer^3^. GOJ cancers of Siewert type I are managed by oesophagectomy, but there is variation in the approach for Siewert type II or III tumours, although oesophagectomy is overrepresented in Siewert type II cancer and total gastrectomy is often preferred in Siewert type III cancer^4^. Additionally, the exact origin of GOJ tumours can sometimes be difficult to accurately assess, particularly in large and bulky tumours, which can make the choice between oesophagectomy and gastrectomy arbitrary^5^. Non-radical resection margins (with cancer involvement) have a profound negative effect on GOJ cancer survival^1^. Studies have shown contradictory results regarding radical (R0) resection rates between gastrectomy and oesophagectomy. Some studies have found no difference between the approaches, while others have shown favourable results for either gastrectomy or oesophagectomy^6–13^. Some research has shown a survival benefit associated with more extensive lymphadenectomy for oesophageal and GOJ cancer^14,15^, while more recent studies have questioned such a benefit after adjustment for confounding, including surgeon volume^16,17^. Nevertheless, the number of removed lymph nodes during surgery is often considered an indicator of the radicality and quality of the surgery. Gastrectomy and oesophagectomy seem to be associated with a similar prognosis^7,18,19^, but uncertainty remains due to the limited number of studies specifically evaluating GOJ cancer. Therefore, we aimed to compare gastrectomy with oesophagectomy in relation to the outcomes radicality of the resection margins, lymph node yield, and survival in patients with GOJ cancer of Siewert type II or III (where gastrectomy and oesophagectomy are alternative surgical approaches), in an unselected cohort study with adjustment for potential confounders.

## Methods

### Study design and data sources

The design of this nationwide Swedish, population-based and prospective cohort study has been described in detail elsewhere^20^. In brief, 90% of all patients who underwent surgery for oesophageal or GOJ cancer in Sweden during the period April 2, 2001 to December 31, 2005 were included. These patients were followed up until February 2016, i.e. we had 5-year follow-up of all patients. Among all 616 patients enrolled in the source cohort, this study included 240 patients who had undergone gastrectomy or oesophagectomy with curative intent for local or locally advanced Siewert type II or III GOJ adenocarcinoma. Siewert II or III tumour was defined based on the postoperative pathology reports as an adenocarcinoma with its epicentre up to 1 cm above or up to 5 cm below the GOJ, measured from the proximal margin of the gastric folds^21^. For some patients with type II or III the exact Siewert classification could not be reliably determined based on the pathology reports, and these patients were further grouped as unclear type II or III GOJ cancers. In 2001–2005, neoadjuvant or adjuvant therapy was rarely used in Sweden, which makes this an almost exclusively surgical cohort rather than a multimodality therapy cohort. Information regarding patient and tumour characteristics and surgical details was prospectively collected from all relevant hospitals in Sweden. Tumour staging was based on the postoperative stage defined by the pathologist according to the 6^th^ edition of TNM-classification, which is uniform regarding Siewert II and III tumours and was used during the time of the data collection^22^. Data on comorbidities were obtained from the Swedish Patient Registry, using the most recent and well-validated version of the Charlson Comorbidity Index^23^. The mortality data were retrieved from the Swedish Causes of Death Registry. The study was approved by the Regional Ethical Review Board in Stockholm, Sweden. All methods were carried out in accordance with relevant guidelines and regulations. All participating patients gave informed consent.

### Exposures

The surgical approach was decided mainly based on the experience of the operating surgeon. The study exposure was either of two surgical approaches:

1. Gastrectomy: Total gastrectomy including resection of the distal oesophagus through laparotomy and anastomosis above the diaphragm. Reconstruction was achieved most commonly using the Roux-en-Y method.
2. Oesophagectomy: Resection of most parts of the oesophagus and the proximal stomach through laparotomy and thoracotomy, with a gastric pull-up reconstruction and an anastomosis in the upper chest in over 95% of the cases.

No consensus regarding the extent of lymphadenectomy existed during the study period, but moderately extensive lymphadenectomies were preferred.

### Outcomes

The primary outcome of the study was tumour involvement of the resection margins: Growth of tumour cells at the cut margin of resection seen under the microscope (R1) or residual tumour that could not be resected during surgery, as reported by the surgeon (R2).

The secondary outcomes of the study were 1) Lymph node yield: The number of lymph nodes removed and identified by the pathologist after the resection; and 2) mortality: The 5-year all-cause mortality, counted from the date of surgery. The crude 5-year mortality analysis has been reported as a secondary outcome of a previous study from this cohort^24^.

### Statistical analysis

Different statistical methods were used for each of the three outcomes under investigation.

1. Multivariable logistic regression was used to evaluate the risk of non-radical resection margins. The risk estimates were odds ratios (ORs) and 95% confidence intervals (CIs), adjusted for the following potential confounding variables: age (categorized into <65 years, 65–75 years, or >75 years), sex (male or female), comorbidity (Charlson’s Comorbidity Index^23,25^ score 0, 1 or ≥2), pathological tumour stage (0-I, II, or III-IV), and surgeon volume (0–3, 4–10, or ≥11 operations during the study period).
2. ANCOVA was used to assess the mean lymph node yield as a continuous variable and to adjust for all confounding factors (with the same categorizations) listed above. The results were presented as mean number of lymph nodes with standard deviation (SD), and mean difference ± SD.
3. Cox proportional hazards model was used to calculate the risk of 5-year mortality. The risk estimates were hazard ratios (HRs) with 95% CIs, adjusted for all confounding factors (with the same categorizations) listed above.

The oesophagectomy group was the reference category in all analyses.

The data management and statistical analyses were conducted by a senior biostatistician (KW), who followed the analysis plan decided in a detailed and pre-defined study protocol. The statistical software IBM SPSS v24.0 (IBM Corp., Armonk, NY) was used for all statistical analyses.

## Results

### Patients

Among all 240 patients with GOJ cancer included in this study, 159 (66%) had Siewert type II cancer, 67 (28%) had Siewert type III cancer and 13 (6%) patients had a type II or III cancer that could not be reliably determined as either. Of these participating patients, 85 (35%) underwent gastrectomy and 155 (65%) underwent oesophagectomy. Patients who underwent gastrectomy were slightly older, had more comorbidity, were more likely to have Siewert type III cancer, low histological grade of differentiation and were less likely to be operated on by high-volume surgeons, while the sex and tumour stage distribution were similar between the comparison groups (Table 1).

**Table 1:** Patient and surgical characteristics of the 240 patients who underwent oesophagectomy or gastrectomy for gastro-oesophageal junction adenocarcinoma of Siewert type II or III.

	Oesophagectomy	Gastrectomy	Total
	Number (%)	Number (%)	Number (%)
**Total**	155 (100)	85 (100)	240 (100)
**Age (in years)**			
<65	66 (42)	27 (32)	93 (39)
65–75	58 (37)	32 (38)	90 (38)
>75	31 (20)	25 (31)	57 (24)
**Sex**			
Male	128 (83)	69 (81)	230 (82)
Female	27 (17)	16 (19)	52 (18)
**Charlson’s Comorbidity Index**			
0	83 (54)	42 (49)	125 (52)
1	40 (26)	17 (20)	57 (24)
≥2	32 (21)	26 (31)	58 (24)
**Siewert type**			
II	122 (79)	37 (44)	159 (66)
III	20 (13)	47 (56)	67 (28)
Unclear II or III	13 (8)	1 (1)	14 (6)
**Histological grade of differentiation**			
Well differentiated	5 (6)	10 (7)	15 (6)
Moderately differentiated	26 (31)	63 (41)	89 (37)
Poorly differentiated	51 (60)	71 (46)	122 (51)
Unknown	3 (4)	11 (7)	14 (6)
**Tumour stage**			
0-I	29 (19)	19 (22)	48 (20)
II	38 (25)	27 (32)	65 (27)
III-IV	83 (54)	39 (46)	122 (51)
Missing	5 (3)	0 (0)	5 (2)
**Surgeon volume**			
Low (≤3)	52 (34)	33 (39)	85 (35)
Mid (4–10)	45 (29)	39 (46)	84 (35)
High (≥11)	58 (37)	13 (15)	71 (30)
**Resection margins**			
R0	134 (87)	72 (85)	206 (86)
R1/R2	21 (14)	13 (15)	34 (14)
**Number of removed lymph nodes**			
≤9	56 (36)	31 (36)	87 (36)
10–17	49 (32)	25 (29)	74 (31)
≥18	47 (30)	25 (29)	72 (30)
Missing	3 (2)	4 (5)	7 (3)

### Resection margins

The proportion of patients who had non-radical resection was similar when comparing gastrectomy (15%) and oesophagectomy (14%). The adjusted OR was not statistically significantly increased when comparing gastrectomy with oesophagectomy (OR 1.61, 95% CI 0.68–3.83) (Table 2).

**Table 2:** Logistic regression analysis of the risk of non-radical resection margin status presented as odds ratios (ORs) and 95% confidence intervals (CIs).

Model	Surgical approach	
	Oesophagectomy n = 155	Gastrectomy n = 85
Crude OR (95% CI)	1 (reference)	1.15 (0.55–2.44)
Adjusted OR (95% CI)^†^	1 (reference)	1.61 (0.68–3.83)

^†^Adjusted for: age, sex, comorbidities, tumour stage and surgeon volume. Five patients excluded from the oesophagectomy group because of missing tumour stage.

### Lymph node yield

The mean number of lymph nodes was similar in the gastrectomy (14.2 nodes, SD ± 9.6) and oesophagectomy (14.2 nodes, SD ± 10.4) groups (p = 0.996) (Table 3). In the adjusted analysis, the number of removed lymph nodes was not statistically increased in the gastrectomy group versus the oesophagectomy group (mean difference 2.4, 95% CI-0.2–5.0).

**Table 3:** Analysis of covariance (ANCOVA) on the number of lymph nodes removed and surgical approach, presented as means with 95% confidence intervals (CIs).

	Surgical approach	
	Oesophagectomy n = 152	Gastrectomy n = 81
Mean (95% CI)	14.2 (12.6–15.8)	14.2 (12.0–16.4)
Adjusted mean (95% CI)^†^	12.7 (10.8–14.7)	15.1 (12.7–17.5)

^†^Adjusted for: age, sex, comorbidities, tumour stage and surgeon volume. Four patients excluded from the oesophagectomy group because of missing tumour stage.

### Mortality

No difference was observed in the absolute 5-year mortality between the gastrectomy (77%) and oesophagectomy (76%) groups. The adjusted HR of all-cause 5-year mortality was 1.07 (95% CI 0.78–1.47) when comparing gastrectomy with oesophagectomy (Table 4).

**Table 4:** Cox proportional-hazards regression analysis of 5-year mortality and surgical approach, presented as hazard ratios (HRs) and 95% confidence intervals (CIs).

	Surgical approach	
	Oesophagectomy n = 155	Gastrectomy n = 85
Crude HR (95% CI)	1 (reference)	1.04 (0.77–1.41)
Adjusted HR (95% CI)^†^	1 (reference)	1.07 (0.78–1.47)

^†^Adjusted for: age, sex, comorbidities, tumour stage and surgeon volume. Five patients excluded from the oesophagectomy group because of missing tumour stage.

## Discussion

This study indicates that gastrectomy and oesophagectomy produce similar outcomes regarding non-radical resection margins, lymph node yield, and 5-year prognosis for Siewert II and III GOJ cancer.

An advantage of this study is the population-based cohort design with coverage of more than 90% of GOJ cancer patients undergoing surgery in Sweden during the study period, providing an unselected cohort. Another strength is the strict inclusion of only Siewert type II and III tumours, where both gastrectomy and oesophagectomy may be used. The prospective data collection and centralized review of these data provided accurate and unbiased information about exposures, outcomes, and confounders. The study followed a detailed study protocol, with all definitions, categorizations, and analyses decided on beforehand. Additionally, the results were adjusted for a number of potential confounders, including surgeon volume^26^. The study period is not very recent, which might be seen as a limitation. However, this made it possible to examine a surgical cohort, almost without influence of neoadjuvant therapy (only 2% in this study), while only more recently the use of neoadjuvant therapy has become standard care. Neoadjuvant therapy could otherwise confound the assessment of surgical approach in relation to all study outcomes in this study. Despite the nationwide assessment of cases, a weakness is the limited sample size followed by the selection of Siewert type II and III tumours. In Sweden the incidence of GOJ cancers is relatively low^27^ and only a small proportion of these patients undergo resection^28^. Yet, this is one of the largest studies comparing gastrectomy and oesophagectomy in relation to resection margins and the extent of lymphadenectomy in Siewert II-III GOJ cancers. Finally, another potential limitation is residual confounding due to factors that we grouped or did not take into account, such as tumour recurrences. However, tumor recurrence data were not available for this study, and we adjusted for all other factors that were considered potential confounders.

This study showed no major difference in the radical resection rates for GOJ in cancer between gastrectomy and oesophagectomy. Only one population-based study and a few single-centre studies have compared rates of radical resection margin between these two approaches in GOJ cancers. Four of these studies support the results of the present study, and the R0 resection rates in this study (85%-86%) are well in line with other previous studies showing R0 resection rates between 72%–93% for oesophagectomy or gastrectomy for GOJ cancer^7,10^. A recent large (n = 1196) population-based Dutch study implied similar radical (R0) resection rates between gastrectomy (84%) and oesophagectomy (87%)^10^. However, this study had a high proportion (41%) of missing T stage data for patients who underwent gastrectomy, and was potentially confounded by the use of neoadjuvant therapy. A single-institution US study of 505 patients with GOJ cancers (393 with Siewert II-III cancer) found no differences in resection margin status between gastrectomy and oesophagectomy^6^. However, surgical treatment evolved during the long duration of that study (1985–2003), for example, 138 of 153 patients in the gastrectomy group underwent proximal gastrectomy, which is no longer used in cancer surgery. A single-centre study from Sweden (n = 143) also suggested similar R0 resection rates for the surgical approaches under study^8^. Finally, a prospective study from Germany examining Siewert II cancer (n = 92) indicated that R0 resection rates were similar between gastrectomy and oesophagectomy^13^. Some studies are in disagreement with the results of the present investigation. A retrospective French study implied a favourable rate of R0 resection with gastrectomy versus oesophagectomy in Siewert I-III GOJ cancer (n = 126)^11^. On the other hand, a retrospective US study found favourable results with oesophagectomy compared to gastrectomy in Siewert type II-III GOJ cancer (n = 82)^9^. Additionally, a prospective Dutch study suggested an increased rate of positive circumferential resection margins associated with gastrectomy in Siewert II patients (n = 176)^12^. Unfortunately, none of these previous studies adjusted their results for tumour stage or surgeon volume. Additionally, the debate of whether to advocate gastrectomy or oesophagectomy is related to Siewert II-III GOJ cancers rather than Siewert I GOJ cancer, for which oesophagectomy is clearly preferred. Inclusion of Siewert I GOJ cancers in some of these aforementioned studies might have produced results that are biased towards favouring oesophagectomy. Taken together, the results of these studies suggest that both gastrectomy and oesophagectomy can be applied to Siewert II-III GOJ cancers from the perspective of tumour-free resection margins.

The present study found similar lymph node yield when comparing gastrectomy and oesophagectomy, which remained after adjustment for surgeon volume and other potential confounding factors. The mean number of lymph nodes removed differ from study to study comparing gastrectomy and oesophagectomy for GOJ cancer, highlighting the differences between institutional treatment regimens^6,9,13^. In the present study, the mean lymph node yield was relatively small with higher variability than other studies on the topic, reflecting the lack of national consensus on the extent of lymphadenectomy in GOJ cancer, as well as the preference for limited lymphadenectomy among upper gastrointestinal surgeons in Sweden. However, five studies, two from the United States^6,9^, one from Germany^13^, and two from the Netherlands^10,12^, found no differences in lymph node yield between gastrectomy and oesophagectomy, which corroborate the findings of the present study. However, surgeon experience has been shown to influence the number of resected lymph nodes during surgery with increasing lymph node counts by increasing surgeon volume^26^, but was adjusted for only in the present study. Nevertheless, taking the results from previous studies and this study into account, gastrectomy and oesophagectomy seem to allow for a similar number of lymph nodes to be removed.

The 5-year all-cause mortality rate observed in this study were similar between the surgical approaches, which is well in line with the results from most previous research on this topic^9–11,19,29^. Taken together, there might not be survival difference between gastrectomy and oesophagectomy in Siewert II and III GOJ cancers, or any difference is likely to be small. As there were no data available on the recurrences in this cohort, it would be interesting to compare the recurrence rates between these approaches, or to conduct analysis by adjusting for the treatment of the recurrences in a larger study.

There are clinical implications to the findings of this study. The results indicate that gastrectomy and oesophagectomy are equally good at achieving complete resection of the tumour and adequate lymphadenectomy without compromising survival in Siewert II and III GOJ cancers, ultimately supporting whichever surgical approach is preferred by the individual surgeon. However, the results also highlight that patient-reported outcomes might be of great relevance in future research comparing gastrectomy and oesophagectomy. Gastrectomy might, for example, reduce the risk of pulmonary complications, which are known to influence the patients’ health-related quality of life in the long term^30^, while gastrectomy might be associated with more nutritional problems.

In conclusion, this population-based cohort study with adjustment for potentially important confounding factors indicates that total gastrectomy and sub-total oesophagectomy provide similar rates of radical resection margins, number of lymph nodes removed, and 5-year all-cause mortality for Siewert II or III GOJ cancer. The surgical approach for Siewert II and III cancers could be chosen on the basis of experience of the surgeon, but gastrectomy might be preferable to avoid thoracotomy-related complications.

## References

[1] Feith M, Stein HJ, Siewert JR (2006). Adenocarcinoma of the esophagogastric junction: surgical therapy based on 1602 consecutive resected patients. Surg Oncol Clin N Am.

[2] Vallbohmer D (2010). A multicenter study of survival after neoadjuvant radiotherapy/chemotherapy and esophagectomy for ypT0N0M0R0 esophageal cancer. Ann Surg.

[3] Kauppila JH, Lagergren J (2016). The surgical management of esophago-gastric junctional cancer. Surg Oncol.

[4] de Manzoni G (2002). Results of surgical treatment of adenocarcinoma of the gastric cardia. Ann Thorac Surg.

[5] Al-Haddad S (2014). Adenocarcinoma at the gastroesophageal junction. Ann N Y Acad Sci.

[6] Barbour AP (2007). Adenocarcinoma of the gastroesophageal junction: influence of esophageal resection margin and operative approach on outcome. Ann Surg.

[7] Haverkamp L, Ruurda JP, van Leeuwen MS, Siersema PD, van Hillegersberg R (2014). Systematic review of the surgical strategies of adenocarcinomas of the gastroesophageal junction. Surg Oncol.

[8] Johansson J (2008). Two different surgical approaches in the treatment of adenocarcinoma at the gastroesophageal junction. World J Surg.

[9] Ito H (2004). Adenocarcinoma of the gastric cardia: what is the optimal surgical approach?. J Am Coll Surg.

[10] Koeter M (2016). Perioperative Treatment, Not Surgical Approach, Influences Overall Survival in Patients with Gastroesophageal Junction Tumors: A Nationwide, Population-Based Study in The Netherlands. Ann Surg Oncol.

[11] Mariette C (2002). Surgical management of and long-term survival after adenocarcinoma of the cardia. Br J Surg.

[12] Parry K (2015). Surgical treatment of adenocarcinomas of the gastro-esophageal junction. Ann Surg Oncol.

[13] Reeh M (2012). Staging and outcome depending on surgical treatment in adenocarcinomas of the oesophagogastric junction. Br J Surg.

[14] Peyre CG (2008). The number of lymph nodes removed predicts survival in esophageal cancer: an international study on the impact of extent of surgical resection. Ann Surg.

[15] Schwarz, R. E. & Smith, D. D. Clinical impact of lymphadenectomy extent in resectable esophageal cancer. *J Gastrointest Surg* **11**, 1384–1393; discussion 1393–1384, 10.1007/s11605-007-0264-2 (2007). 10.1007/s11605-007-0264-2 17764019

[16] van der Schaaf, M., Johar, A., Wijnhoven, B., Lagergren, P. & Lagergren, J. Extent of lymph node removal during esophageal cancer surgery and survival. *J Natl Cancer Inst* **107**, 10.1093/jnci/djv043 (2015). 10.1093/jnci/djv043 25748792

[17] Lagergren J (2016). Extent of Lymphadenectomy and Prognosis After Esophageal Cancer Surgery. JAMA Surg.

[18] Wei MT (2014). Transthoracic vs transhiatal surgery for cancer of the esophagogastric junction: a meta-analysis. World J Gastroenterol.

[19] Martin JT, Mahan A, Zwischenberger JB, McGrath PC, Tzeng CW (2015). Should gastric cardia cancers be treated with esophagectomy or total gastrectomy? A comprehensive analysis of 4,996 NSQIP/SEER patients. J Am Coll Surg.

[20] Derogar M, Lagergren P (2012). Health-related quality of life among 5-year survivors of esophageal cancer surgery: a prospective population-based study. J Clin Oncol.

[21] Siewert JR, Stein HJ (1998). Classification of adenocarcinoma of the oesophagogastric junction. Br J Surg.

[22] Sobin, L. H., Wittekind, C. & International Union against Cancer. *TNM: classification of malignant tumours*. 6th edn, (Wiley-Liss, 2002).

[23] Armitage JN, van der Meulen JH, Royal College of Surgeons Co-morbidity Consensus, G (2010). Identifying co-morbidity in surgical patients using administrative data with the Royal College of Surgeons Charlson Score. Br J Surg.

[24] Kauppila, J. H., Ringborg, C., Johar, A., Lagergren, J. & Lagergren, P. Health-related quality of life after gastrectomy, esophagectomy, and combined esophagogastrectomy for gastroesophageal junction adenocarcinoma. *Gastric Cancer*, 10.1007/s10120-017-0761-2 (2017). 10.1007/s10120-017-0761-2 PMC5906505 28852939

[25] Charlson ME, Pompei P, Ales KL, MacKenzie CR (1987). A new method of classifying prognostic comorbidity in longitudinal studies: development and validation. J Chronic Dis.

[26] Markar SR, Mackenzie H, Lagergren P, Hanna GB, Lagergren J (2016). Surgical Proficiency Gain and Survival After Esophagectomy for Cancer. J Clin Oncol.

[27] Lagergren J, Mattsson F (2011). No further increase in the incidence of esophageal adenocarcinoma in Sweden. Int J Cancer.

[28] Rouvelas, I. *et al*. Impact of hospital volume on long-term survival after esophageal cancer surgery. *Arch Surg* **142**, 113–117; discussion 118, 10.1001/archsurg.142.2.113 (2007). 10.1001/archsurg.142.2.113 17309961

[29] Rudiger Siewert J, Feith M, Werner M, Stein HJ (2000). Adenocarcinoma of the esophagogastric junction: results of surgical therapy based on anatomical/topographic classification in 1,002 consecutive patients. Ann Surg.

[30] Derogar M, Orsini N, Sadr-Azodi O, Lagergren P (2012). Influence of major postoperative complications on health-related quality of life among long-term survivors of esophageal cancer surgery. J Clin Oncol.

